# A cooling off period: decline in the use of hot biopsy forceps technique in colonoscopy in the U.S. Medicare population 2000–2019

**DOI:** 10.1186/s12876-025-04020-9

**Published:** 2025-05-27

**Authors:** Andrew J. Read, Jacob E. Kurlander, Akbar K. Waljee, Sameer D. Saini

**Affiliations:** 1 Division of Gastroenterology, Department of Internal Medicine, University of Michigan, 3912 E Taubman Center 1500 E Medical Center Dr., SPC 2435, MI 48109 Ann Arbor, USA; 2 Institute for Healthcare Policy and Innovation, University of Michigan, Ann Arbor, MI USA; 3 Rogel Cancer Center, University of Michigan, Ann Arbor, MI USA; 4 VA HSR&D Center for Clinical Management Research, Ann Arbor, MI USA; 5 Department of Learning Health Sciences, University of Michigan, Ann Arbor, MI USA

**Keywords:** Colonoscopy, Hot biopsy forceps, Cold biopsy forceps, Snare polypectomy

## Abstract

**Background:**

The use of hot biopsy forceps (with electrocautery) is no longer routinely recommended given increased complications compared to cold biopsy forceps (without electrocautery). It is unknown how often the technique is currently used in the United States (U.S.) or how its usage has changed over time.

**Aim:**

To characterize the use of hot biopsy forceps by U.S. Medicare providers over time, identify provider characteristics of those who more commonly perform this technique, and determine if there are regional differences in use of this technique within the U.S.

**Methods:**

We performed a retrospective cross-sectional study using U.S. Medicare summary data from 2000 to 2019 to analyze the frequency of cold and hot biopsies. We used detailed provider and state summary files to characterize providers’ demographics, including geographic region, to identify regional variation in use of these techniques, and identify factors associated with use of hot biopsy forceps from 2012 to 2019.

**Results:**

The hot biopsy forceps technique peaked in 2003 (412,165/year) and declined to 108,232/year in 2019, while the cold biopsy forceps technique increased from 482,862/year in 2000 to 1,533,558/year in 2019. Use of hot biopsy forceps was more common by non-gastroenterologists and in rural practice settings. In addition, there was up to 50-fold difference in utilization in these techniques between states (on a population normalized basis), with the highest rate of use in the southeastern U.S.

**Conclusion:**

Variation in the use of hot biopsy forceps by region and provider suggests a potential area for quality improvement given the comparative advantages of the cold biopsy forceps technique. De-implementation of an existing endoscopic practice may require different approaches than implementation of a new practice.

## Introduction

Complete resection of adenomatous colon polyps is essential to disrupt the adenoma-to-carcinoma sequence and prevent the development of colorectal cancer [1]. Multiple polypectomy tools are available, including biopsy forceps (for smaller polyps) or snares (for larger lesions), and resection can be achieved using electrocautery (hot) or without electrocautery (cold) [2]. While hot biopsy forceps (using electrocautery) were once commonly used for resection of smaller colon polyps, data suggest this technique increases the risks of thermal injury, delayed bleeding, and decreased histopathology specimen quality compared to cold biopsy forceps (without electrocautery) [3–7]. In addition, forceps techniques have been associated with increased risk for incomplete polyp resection compared to snare polypectomy techniques for larger polyps [8, 9].

Potential complications associated with hot biopsy forceps were described in an American Society for Gastrointestinal Endoscopy (ASGE) technology assessment guidance in 1992 [4], and a subsequent update in 2006 advised that hot biopsies were “not recommended for routine tissue sampling” [10]. More recent guidelines have more strongly recommended against the use of these techniques, including 2017 guidance from the European Society for Gastrointestinal Endoscopy, recommending against using the hot biopsy forceps technique [11]. Similarly, in a 2020 guideline, the U.S. Multi-Society Task Force on Colorectal Cancer recommended against the routine use of hot biopsy forceps for diminutive and small lesions [12]. However, there are limited data on the current use of hot biopsy forceps in the U.S. or changes in use of the technique over time.

Our objective was to understand the use of hot and cold biopsy forceps techniques by U.S. Medicare providers over time, describe the characteristics of providers performing hot and cold biopsies, determine potential regional differences in use of these techniques, and identify factors associated with the use of hot biopsy forceps.

## Methods

### Study design

We conducted a retrospective cross-sectional evaluation of administrative data from Medicare Part B from 2000 to 2019 for national trends, and from 2012 to 2019, for individual provider-level and state-level data. We hypothesized that the use of hot biopsy forceps would decline over time with a concurrent rise in the use of cold biopsy forceps, but that the use of hot biopsy forceps would nonetheless persist in some providers due to ingrained practice patterns. To test this hypothesis, we examined the number of biopsies performed per year using hot and cold biopsy forceps techniques. We evaluated demographic covariates including the provider type (e.g., gastroenterologist, surgeon, etc.), provider’s gender, and geography of the practice setting.

This study was considered exempt and not regulated by the University of Michigan Institutional Review Board (HUM00173993) as all data are publicly available and without patient-level identifiers (per U.S. Code of Federal Regulations 45 CFR Part 46). As this was a retrospective analysis of a publicly available dataset, without any individual patient-level identifiers, informed consent was deemed unnecessary per national regulations (U.S. Code of Federal Regulations 45 CFR Part 46).

### Population

Data from publicly available Medicare Part B datasets and Medicare enrollment were obtained from the Centers for Medicare & Medicaid Services (CMS) website (CMS.gov). Three publicly available Medicare Part B datasets (CMS) were analyzed: (1) annual national summary files from 2000 to 2019 with total procedures performed for colonoscopy with hot and cold biopsy forceps; (2) state summary files containing totals by state per year (2012–2019); and (3) individual provider-level data for providers who performed more than 10 of an individual procedure per year (2012–2019).

We used the annual national Medicare Part B summary data to determine the number of hot and cold colonoscopy biopsy procedures performed per year by the corresponding Current Procedural Terminology (CPT) codes: CPT 45384 for hot biopsy forceps and CPT 45380 for cold biopsy forceps.

We used the annual Medicare Part B state summary files (2012–2019) to assess for potential regional or geographic differences in the use of hot and cold biopsy forceps in colonoscopies. We used each state’s annual Medicare enrollment data to calculate an adjusted rate of use of each technique by state per year (e.g., number of procedures with hot biopsies performed / 10,000 Medicare enrollees per year). This allowed us to control for changes in Medicare enrollment over time within states, and allowed for comparisons between states with different populations.

### Statistical analysis

We calculated descriptive statistics for endoscopists performing colonoscopies with hot and/or cold biopsies using the national Medicare Part B individual provider level files (2012–2019). The individual provider file includes data on providers who performed more than 10 of an individual Current Procedural Terminology (CPT) procedure code in a year. (Data were not available for providers who performed 10 or fewer of an individual procedure type.) We linked this to the Medicare Part B file by NPI (National Provider Identifier) numbers to improve characterization of the providers’ demographics, including each provider’s gender (as self-reported male/female in NPI profile), date of NPI number enumeration, specialty information by taxonomy codes, and practice street address. As provider age or length of clinical practice is not explicitly available within these datasets, we calculated an approximate duration of clinical practice by the difference between the year of evaluation (e.g., 2019) and the year of NPI number enumeration. We used the Centers for Disease Control and Prevention (CDC) National Center for Health Statistics 2013 (NCHS) Urban-Rural Classification Scheme for Counties to classify each provider’s practice environment by degree of urbanization based on each provider’s address [13].

We determined each provider’s medical specialty using the provider type listed within the Medicare Part B file and, in cases of ambiguity, used the detailed healthcare provider taxonomy codes from the National Plan and Provider Enumeration System (NPPES) file (linked by NPI numbers) to further improve specificity. For example, in cases of providers listed as “Internal Medicine” providers, we further defined them as gastroenterologists if their taxonomy codes were “207RG0100X” (gastroenterology), “207RI0008X” (hepatology), or “207RT0003X” (transplant hepatology). In addition, we characterized endoscopists’ clinical practice pattern by examining other procedure codes performed by those providers. For example, we categorized providers as advanced endoscopists if they performed more than 10 endoscopic retrograde cholangiopancreatography (ERCP) or endoscopic ultrasound (EUS) procedures within the year, using the corresponding CPT codes. We also determined if endoscopists also performed esophagogastroduodenoscopy (EGD) procedures using the corresponding CPT codes. We used a multivariable logistic regression model to calculate the odds ratio of the association of providers’ demographic variables and use of hot biopsy forceps technique in a single year (2019).

Statistical analysis was performed using SAS 9.4 (SAS Institute, Cary, NC) and R Core Team (2023). Graphs were produced using GraphPad Prism 8.0 (GraphPad Software, San Diego, CA) and maps using R Core Team (2023).

## Results

### Provider characteristics

We characterized the demographics and practice locations of individual providers performing colonoscopies with more than 10 hot or cold biopsies per year by gender, practice setting (size of community by National Center for Health Statistics [NCHS] classification), estimated years in clinical practice (based on NPI enumeration date), and specialty type (Table 1). We identified 15,435 providers who performed more than 10 colonoscopies with hot or cold biopsies in 2019 (Table 1). We analyzed the subsets of providers who performed more than 10 hot biopsies (*n* = 1,409) and those who performed more than 10 cold biopsies (*n* = 14,026) but not hot biopsies. Billing codes associated with ambulatory surgery centers (ASCs) were not included in this analysis, as the individual provider type was unknown.

**Table 1:** Demographic and practice characteristics of providers performing > 10 colonoscopy with biopsies in Medicare Part B in 2019, as defined by > 10 cold biopsies (CPT 45380) or > 10 hot biopsies (CPT 45384) in 2019. Percentages shown represent column percentages by category. (EGD = Esophagogastroduodenoscopy; ERCP = Endoscopic retrograde cholangiopancreatography; EUS = Endoscopic Ultrasound); NCHS = National Center for Health Statistics)

Characteristic	Providers performing colonoscopy	Providers performing hot biopsy	Providers performing cold biopsy only
**Provider Specialty no. (%)**			
Gastroenterology	12,225 (79.2%)	649 (46.1%)	11,576 (82.5%)
Surgery	2,693 (17.5%)	643 (45.6%)	2,050 (14.6%)
Internal / Family Medicine	463 (3.0%)	104 (7.38%)	359 (2.6%)
Other	54 (0.4%)	13 (0.9%)	41 (0.3%)
Total	*N* = 15,435	*N* = 1,409	*N* = 14,026
**Practice Duration no. (%)**			
< 10 years	1,723 (11.2%)	52 (3.7%)	1,671 (11.9%)
10 or more years	13,712 (88.8%)	1,357 (96.3%)	12,355 (88.1%)
**Gender no. (%)**			
Male	12,785 (82.8%)	1,300 (92.3%)	11,485 (81.9%)
Female	2,650 (17.2%)	109 (7.7%)	2,541 (18.1%)
**Clinical Practice Pattern no. (%)**			
Performs EGD	13,955 (90.4%)	1,131 (80.3%)	12,824 (91.4%)
Advanced Endoscopy (ERCP)	1,460 (9.5%)	74 (5.3%)	1,386 (9.9%)
Advanced Endoscopy (EUS)	1,133 (7.3%)	18 (1.3%)	1,115 (8.0%)
**Biopsies Performed by Provider**	All biopsies	Hot biopsies	Cold biopsies
Median	51	29	51
IQR	27–93	18–58	26–91
Maximum	820	566	664
**Practice Setting (NCHS Classification) no. (%)**			
**Metropolitan**			
1. Large central metro (Urban)	5,086 (33.0%)	224 (15.9%)	4,862 (34.7%)
2. Large fringe metro	3,495 (22.6%)	287 (20.4%)	3,208 (22.9%)
3. Medium metro	3,389 (22.0%)	311 (22.1%)	3,078 (21.9%)
4. Small metro	1,716 (11.1%)	182 (12.9%)	1,534 (10.9%)
**Non-Metropolitan**			
5. Micropolitan	1,319 (8.6%)	276 (19.6%)	1,043 (7.4%)
6. Non-core (Rural)	430 (2.8%)	129 (9.2%)	301 (2.2%)

Gastroenterologists performed the largest share of colonoscopies with biopsies (*n* = 12,225 / 15,435, 79.2%), followed by surgeons (*n* = 2,693 / 15,435, 17.5%). Fewer than 10% of the providers were advanced endoscopists, with 9.5% (*n* = 1,460 / 15,435) performing ERCP and 7.3% (*n* = 1,133 / 15,435) performing EUS. More than half of the providers practiced in higher density population regions, with 55.6% (*n* = 8,581 / 15,435) in large central metro or large fringe metro areas (Table 1).

### Use of hot biopsy forceps technique

We examined the provider characteristics as predictors of performing hot biopsy using multivariable logistic regression for the year 2019 (Table 2). Provider characteristics associated with increased use of hot biopsies included provider type (for surgeon vs. gastroenterologist, OR = 4.784, 95% CI 4.180–5.475, *p* <.0001; for internal / family medicine vs. gastroenterologist, OR = 3.817, 95% CI 2.973–4.902, *p* <.0001; other vs. gastroenterologist, OR = 4.831, 95% CI 2.529–9.229, *p* <.0001), male providers (OR = 2.098, 95% CI 1.704–2.583, *p* <.0001), and non-metropolitan regions (OR = 1.64, 95% CI 1.413–1.903, *p* <.0001). Advanced endoscopists were marginally less likely to perform hot biopsies compared to general endoscopists (OR = 0.783, 95% CI 0.618–0.993, *p* =.0434). Increasing years in practice, based on estimated practice length calculated by NPI enumeration date, was also associated with an increased likelihood of performing hot biopsies (OR = 1.308, 95% CI 1.251–1.367, *p* <.0001) (Table 2).

**Table 2:** Multivariable logistic regression model for factors associated with use of hot biopsy forceps technique based on years in practice (continuous variable), gender (binary based on NPI classification), clinical specialty (categorical), advanced endoscopist status (binary, performs ERCP or EUS), urban-rural practice setting (categorical by NCHS classifications of metropolitan for NCHS categories 1–4 and non-metropolitan for NCHS categories 5–6). Logistic regression is based on single year (2019) data

Covariate	Odds Ratio	95% Confidence Interval	*P* value
Years in Practice	1.308	1.251–1.367	< 0.0001
Male	2.098	1.704–2.583	< 0.0001
Advanced endoscopist	0.783	0.618–0.993	0.0434
**Provider Type**			
Gastroenterologist	(Ref.)	(Ref.)	(Ref.)
Surgeon (vs. Gastroenterologist)	4.784	4.180–5.475	< 0.0001
Internal / Family Medicine (vs. Gastroenterologist)	3.817	2.973–4.902	< 0.0001
Other (vs. Gastroenterologist)	4.831	2.529–9.229	< 0.0001
**Urban-Rural**			
Urban (Metropolitan, NCHS 1–4)	(Ref.)	(Ref.)	(Ref.)
Rural (Non-metropolitan, NCHS 5–6)	1.640	1.413–1.903	< 0.0001

### Use of hot biopsy forceps technique over time

**Fig. 1:**
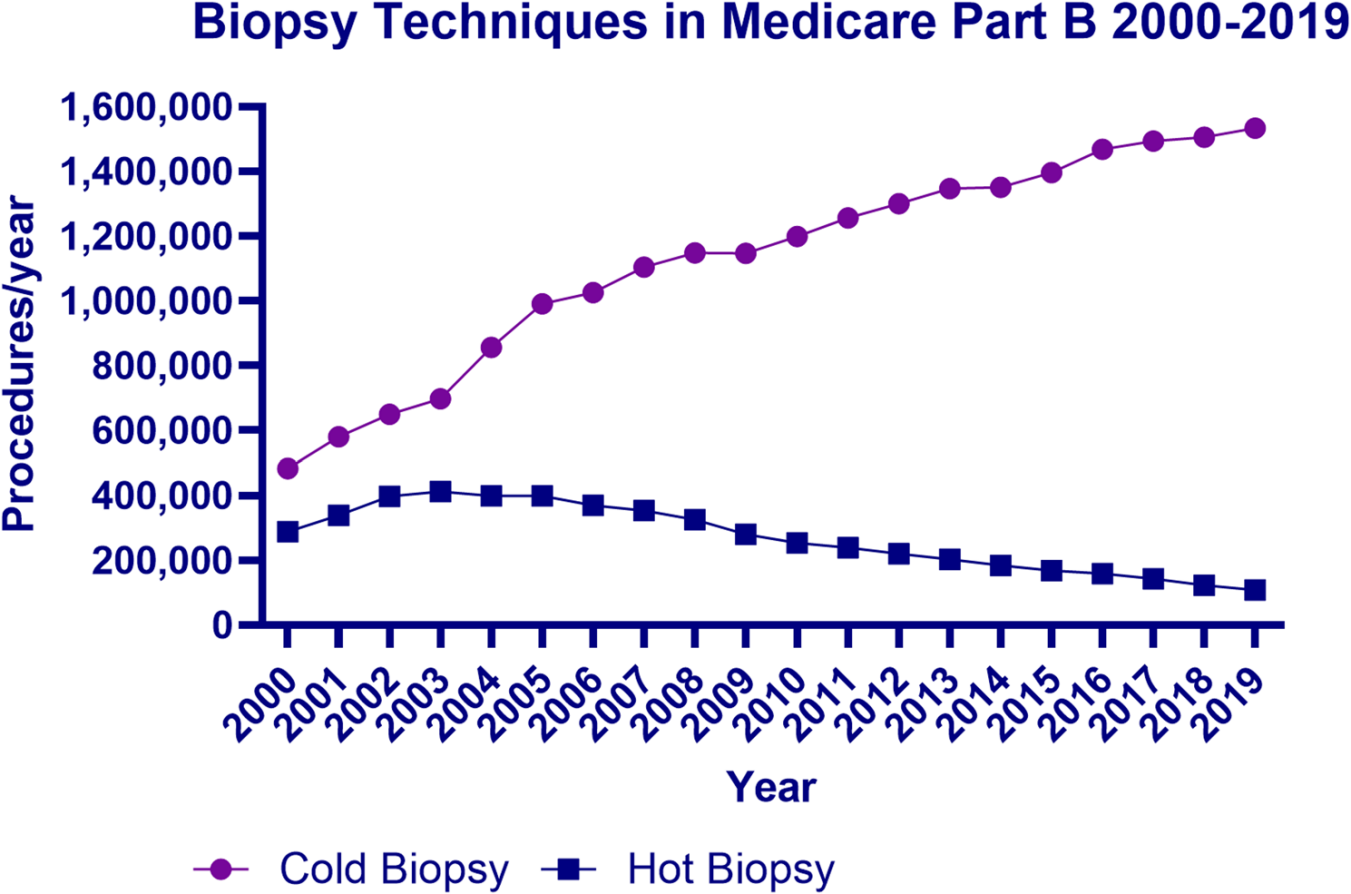
Biopsy techniques in Medicare Part B 2000–2019 (national). Purple (circle) represents the number of cold biopsy procedures per year (CPT 45380). Blue (square) represents the number of hot biopsy procedures per year (CPT 45384)

During the study period (2000–2019), the unadjusted use of hot biopsy forceps increased from 288,493 in 2000, to a peak of 412,165 procedures in 2003, and then subsequently gradually declined to 108,232 in 2019. Simultaneously, the use of cold biopsy forceps technique increased from 482,862 procedures per year in 2000, to 1,533,558 procedures per year in 2019. The proportion of all colonoscopies with biopsies performed with a cold technique increased from 62.6% in 2000 (*n* = 482,862 / 771,355) to 93.4% in 2019 (*n* = 1,533,558 / 1,641,790) (Fig. 1).

Consistent with the national trend, we found a decline in hot biopsy forceps usage by all provider types (gastroenterologists, surgeons, etc.) performing colonoscopy. However, the rate of use of colonoscopy hot biopsy forceps technique remained higher in surgeons compared to gastroenterologists. For example, in 2019, 23.9% of surgeons performing colonoscopy with biopsy (*n* = 643 / 2,693) used hot biopsy forceps technique, versus 37.6% of surgeons performing colonoscopy with biopsy in 2012 (*n* = 1,017 / 2,705). In contrast, only 5.3% of gastroenterologists (*n* = 649 / 12,225) performing colonoscopy with biopsy in 2019 used hot biopsy forceps, a decline from 14.1% of gastroenterologists performing colonoscopy with biopsy in 2012 (*n* = 1,575 / 11,180). Of the 2,792 individual endoscopists who performed the hot biopsy forceps technique in 2012, 822 individuals (29.4%) continued using this technique each year throughout the study period. Among those endoscopists who used hot biopsy forceps each year from 2012 to 2019, the mean number of hot biopsies decreased by 15.7% over this period (from 73.8 to 62.2). For those providers who used hot biopsy forceps in 2019, the median proportion of their total biopsies performed using hot biopsy technique was 0.70 (IQR 0.4-1.0), indicating that most of these providers favored this technique over cold biopsy forceps (Table 3).

**Table 3:** Provider-level analysis in single year (2019), categorized by U.S. Census regions and U.S. Census divisions. GI provider as number and percentage of providers performing colonoscopy with biopsy who are gastroenterologists by region. *Rural provider categorized by practice location as non-metropolitan (NCHS categories 5–6). **Total biopsies/provider is the combined cold and hot biopsies per provider. ***Hot biopsies/provider among those providers performing the hot biopsy forceps technique (CPT 45384). ****Among those providers performing the hot biopsy forceps technique (> 10 in 2019), the proportion of total biopsies per provider that are hot

Census Region Census Division	Endoscopists with biopsies	GI Provider no. (%)	Rural provider (*) no. (%)	Total Biopsies (**) / provider Median (IQR)	Performs hot biopsy no. (%)	Hot biopsies/ provider (***) Median (IQR)	Proportion of biopsies / provider that are hot (****) Median (IQR)
**Region 1** - **Northeast**	3,480	3,093 (88.9%)	194 (5.6%)	47 (26–82)	224 (6.4%)	25.5 (17–50)	0.5 (0.3-1.0)
Division 1 - New England	945	833 (88.2%)	89 (9.4%)	52 (27–87)	28 (3.0%)	31 (17.5–50.5)	0.6 (0.4-1.0)
Division 2 - Middle Atlantic	2,535	2,260 (89.2%)	105 (4.1%)	45 (25–80)	196 (7.7%)	25 (17–50)	0.5 (0.3–0.8)
**Region 2** - **Midwest**	3,646	2,475 (67.9%)	656 (18.0%)	46 (24–82)	372 (10.2%)	30 (18–49)	0.8 (0.5-1.0)
Division 3 - East North Central	2,406	1,729 (71.9%)	342 (14.2%)	48 (25–86)	247 (10.3%)	32 (19–51)	0.7 (0.5-1.0)
Division 4 - West North Central	1,240	746 (60.2%)	314 (25.3%)	40 (21–74)	125 (10.1%)	26 (17–45)	0.9 (0.6-1.0)
**Region 3** - **South**	5,515	4,383 (79.5%)	646 (11.7%)	57 (28–103)	689 (12.5%)	30 (18–64)	0.7 (0.5-1.0)
Division 5 - South Atlantic	3,023	2,515 (83.2%)	236 (7.8%)	60 (30–107)	337 (11.2%)	28 (17–63)	0.7 (0.4-1.0)
Division 6 - East South Central	937	667 (71.2%)	242 (25.8%)	52 (26–101)	178 (19.0%)	29.5 (19–60)	0.7 (0.5-1.0)
Division 7 - West South Central	1,555	1,201 (77.2%)	168 (10.8%)	53 (27–101)	174 (11.1%)	35 (17–71)	0.8 (0.6-1.0)
**Region 4** - **West**	2,794	2,274 (81.4%)	253 (9.1%)	58 (30–101)	124 (4.4%)	32 (16-59.5)	0.6 (0.3-1.0)
Division 8 - Mountain	952	706 (74.2%)	149 (15.7%)	57 (29–100)	37 (3.9%)	21 (12–48)	1.0 (0.4-1.0)
Division 9 - Pacific	1,842	1,568 (85.1%)	104 (5.7%)	48 (30–102)	87 (4.7%)	34 (17–71)	0.6 (0.3–0.9)
**Total**	**15,435**	**12,225** (79.2%)	**1,749** (11.3%)	**51** (27–93)	**1,409** (9.1%)	**29** (18–58)	**0.7** (0.4-1.0)

### Regional variation

**Fig. 2:**
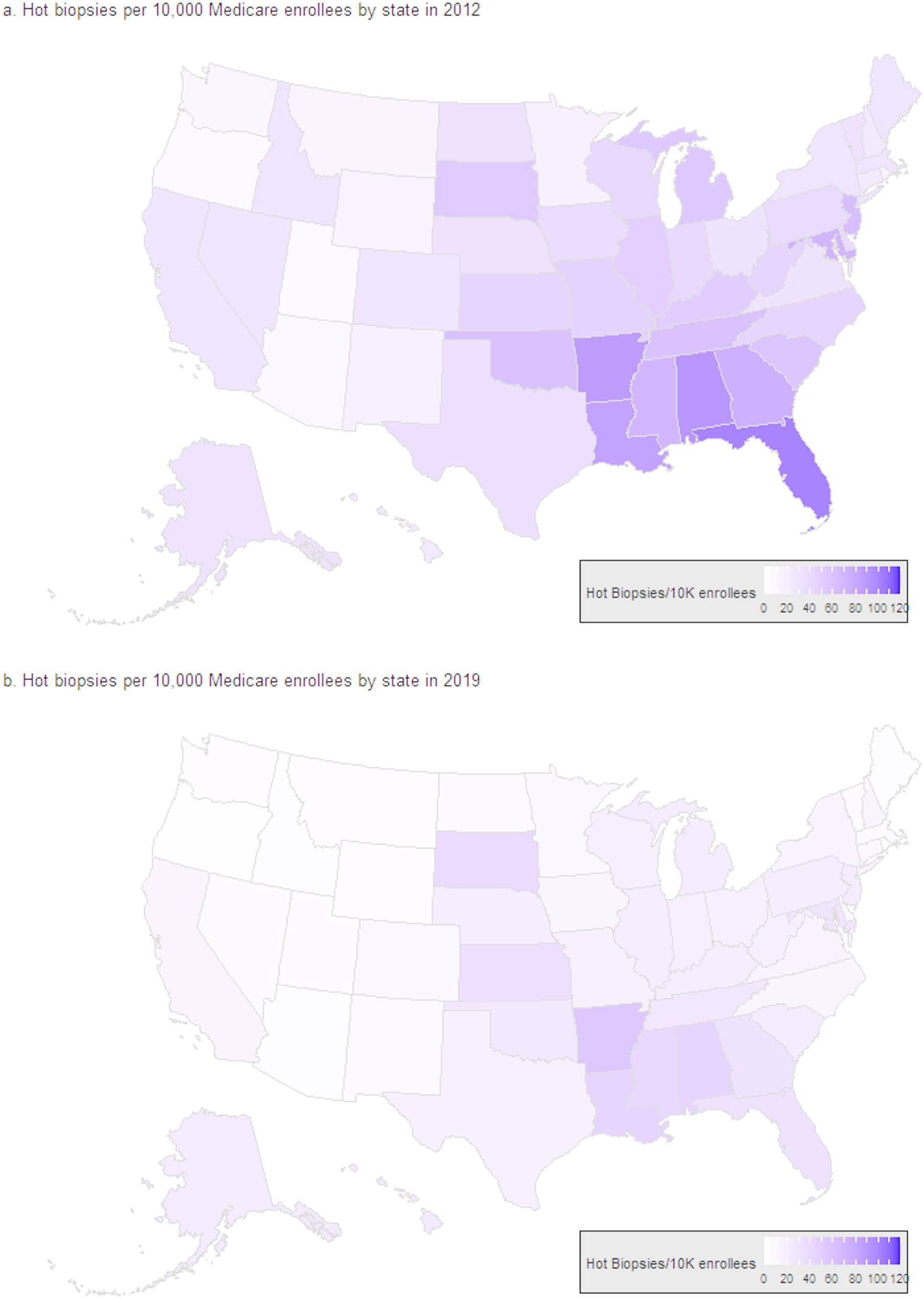
Variation in use of hot biopsy forceps by state in 2012 (**a**) and 2019 (**b**), as rate per 10,000 Medicare enrollees. The rate of hot biopsy forceps was adjusted for annual Medicare enrollees per state by year. Lighter colors indicate lower rates of use of hot biopsy forceps per 10,000 enrollees. Darker colors indicate higher rates of use of hot biopsy forceps

We examined the rate of hot biopsy procedures performed per Medicare enrollees per state on an annual basis (i.e., hot biopsy procedures per 10,000 Medicare enrollees per year). We found that there was increased regional usage of hot biopsy forceps in the southeastern U.S., a trend which was sustained from 2012 to 2019 (Fig. 2). Consistent with the overall national trend (Fig. 1), the use of hot biopsy forceps declined in all states over this period. However, there remained substantial differences between states in the use of hot biopsy forceps. For example, in 2019 in Rhode Island there were 1.04 patients undergoing hot biopsy forceps/10,000 Medicare enrollees vs. 55.7 patients undergoing hot biopsy procedures/10,000 Medicare enrollees in Arkansas, demonstrating an up to 50-fold variation in usage of the technique between states. When the regions were analyzed to better understand these differences, we found the greatest percentages of providers using the hot biopsy forceps technique in the South (12.49%, *n* = 689 / 5,515) and Midwest (10.20%, *n* = 372 / 2,475) (Table 3). These regions also had the most providers who were rural (18.0% in Midwest and 11.7% in South) and the most non-gastroenterologists performing colonoscopy (Table 3).

## Discussion

In the U.S. Medicare population undergoing colonoscopy, we found that the use of hot biopsy forceps has declined over the past decade, with an associated increase in the use of cold biopsy forceps. This trend began in 2003, prior to the 2006 publication of ASGE guidelines recommending against routine sampling with hot biopsy forceps [10]. The nearly parallel decline in hot biopsy forceps and increase in cold biopsy forceps likely reflects growing awareness of the disadvantages of hot biopsy forceps technique and the comparatively superior safety profiles of cold biopsy forceps. Multiple guidelines now recommend against routine use of hot biopsy forceps, and while not reflected in this dataset, the rate may have continued to decline after publication of the most recent U.S. guidelines in 2020 [11, 12]. However, there remains substantial variation in the use of the hot biopsy technique, particularly by endoscopist type (OR = 4.784 for surgeons vs. gastroenterologists). In addition, we found substantial regional variation, with up to a 50-fold differences in utilization of the hot biopsy forceps technique between states. Since the cold technique avoids the potential complications of electrocautery (post-polypectomy bleeding, bowel perforation, and post-polypectomy syndrome), these observed differences are likely to be associated with avoidable patient harms without added benefit.

Previous investigators have shown regional variations among U.S. Medicare providers, with surgeons more likely to perform colonoscopies in rural settings than gastroenterologists [14], and an increased availability of gastroenterologists in urban areas, consistent with our findings [15]. While this dataset does not contain endoscopy quality measures, other studies have identified improved quality measures and outcomes for endoscopies performed by gastroenterologists compared to surgeons [16, 17]. When analyzed by regional differences, we found that areas of increased hot biopsy forceps usage were associated with increased rurality and non-gastroenterologists performing colonoscopy.

Our study suggests several provider-level variables that may contribute to this variation in practice, by examining a specific technique that has low-value. First, an endoscopist’s initial training in gastroenterology vs. surgery may explain some of this variation. Surgeons were more likely to use the hot biopsy forceps technique than gastroenterologists, implying that surgical trainees were more likely to be trained in hot biopsy forceps than GI trainees. The actual trainee procedural volumes and effects of training differences (gastroenterology or surgery) are unknown but are an important topic for future study. We also found that more experienced endoscopists were more likely to use hot biopsy forceps compared to those who had recently completed training. This may reflect the lag from acquiring skills during residency/fellowship training to changes in regular clinical practice: endoscopists likely continue to practice with the techniques they learned as trainees, with senior providers more likely to favor hot biopsy forceps as they were more commonly used previously. In contrast, early career providers who completed training in the past decade, are less likely to have routinely encountered hot biopsy forceps in their training (as use of the procedure was already declining, Fig. 1), and thus less likely to use hot biopsy forceps routinely in practice. More rural areas were associated with increased likelihood of hot biopsy forceps usage (OR = 1.640). Endoscopists in more rural areas are less likely to practice at a large academic medical center and may not have the benefit of as frequent interactions with other endoscopists and may thus be more reluctant to change their current clinical practice. However, there may also be additional local, institutional, or regional training differences or practice preferences that are not captured in this dataset and could be examined in future work.

While this study demonstrates some important variation in clinical practice, there are several limitations to the methodology. First, providers who performed fewer than 11 of a given procedure are not included in the dataset, so it is possible that there are more total providers using hot biopsy forceps (although this would still mean those providers are not commonly using this technique). In addition, as some biopsies were billed under ambulatory surgery center (ASC) national provider identification (NPI) codes, we were unable to further characterize the provider types at those locations (e.g., surgeons vs. gastroenterologists). Similarly, there could be under-capture of individual providers during an inpatient hospitalization/emergency procedure basis. The total absolute number of biopsies per year is also unknown, as multiple biopsies may be performed during a single procedure but would utilize only a single billing code (e.g., 3 cold forceps biopsies would be coded the same as a single cold forceps biopsy). Other contemporaneous trends in endoscopic practices, such as variation in the use of electrocautery for snare polypectomy (i.e., hot vs. cold polypectomy), could not be analyzed by this methodology, as there are not distinct billing codes for snare polypectomy with and without electrocautery. Given similar comparative advantages between cold forceps and cold snare polypectomy, a parallel trend in polypectomy techniques may also coincide with our study period.

Perhaps the primary limitation of the use of an administrative database based on provider-level activity is the absence of patient-level information, such as the indications for the procedures or the specific reasons for selecting one biopsy technique vs. another. As a result, the specific clinical decision-making rationale for individual cases cannot be inferred from this dataset. There may be other unmeasured factors at the patient, provider, institutional or payor level that may influence biopsy technique selection. Although we controlled for Medicare enrollment by state over time to allow for comparisons in utilization of techniques between states, we did not have patient-level data to allow for risk adjustment for comorbidities between states. This study population is limited to U.S. Medicare beneficiaries, who are predominantly aged 65 and older, and thus may not generalize to the non-Medicare population. Most important, this methodology does not allow for comparison of patient-level outcomes between those undergoing hot biopsy forceps vs. cold biopsy forceps, and thus any potential harms are not captured within this dataset. For rare events, such as potential complications related to electrocautery from a hot biopsy forceps, the average provider is unlikely to encounter this in a given year, and thus potential population-level risks may be underappreciated. We did not analyze other potential uses of hot biopsy forceps, which may be used for other therapeutic indications: e.g., avulsion therapy during endoscopic mucosal resection (EMR) lesions or treatment of bleeding [11, 12, 18]. While those indications are more accurately coded under billing codes for treatment of GI bleeding or EMR, it is possible there may also have been miscoding in some cases for these applications.

Nonetheless, despite these limitations, the persistent variation in the use of hot biopsy forceps by region and provider type we identified suggests a potential area for quality improvement given the comparative advantages of cold biopsy forceps vs. hot biopsy forceps. In implementation science, it is often estimated that it takes approximately 17 years for a new practice to be widely implemented in medicine [19, 20]. Identification of practice variation in administrative datasets, such as in this study, may allow for more targeted de-implementation strategies given the relative advantages of cold biopsy forceps, or for non-diminutive polyps, use of snare techniques [21]. Further studies can assess the individual-level barriers to de-implementation and design strategies to target those barriers.

## Conclusion

This study demonstrates a decline but persistent usage of hot biopsy forceps despite comparative advantages of cold biopsy forceps. There is substantial variation in the use of hot biopsy forceps in the U.S. by region, rurality, and provider type (gastroenterologist or surgeon). Given this practice variation, further study of the individual barriers could represent a potential area for quality improvement and de-implementation strategies.

## Data Availability

All data are publicly available from Centers for Medicare and Medicaid Services (https://www.cms.gov/data-research).
